# Pourfour Du Petit Syndrome Due to Ipsilateral Internal Jugular Vein Distention

**DOI:** 10.7759/cureus.37853

**Published:** 2023-04-19

**Authors:** Jaime Leonardo I Salazar-Orellana, R Daniel Aceytuno, Nelson A Vásquez-Cortez, Juan R Umaña-Cerros, Enyis Y Medrano-Machado

**Affiliations:** 1 Department of Neurology, Instituto Salvadoreño del Seguro Social, San Salvador, SLV; 2 Department of Family Medicine, Northern Ontario School of Medicine, Sudbury, CAN; 3 Department of Internal Medicine, Instituto Salvadoreño del Seguro Social, San Salvador, SLV; 4 Department of Radiology, Instituto Salvadoreño del Seguro Social, San Salvador, SLV

**Keywords:** hyperhidrosis, internal jugular vein, sympathetic nervous system, sympathetic chain, pourfour du petit syndrome

## Abstract

Pourfour du Petit Syndrome (PdPS) is characterized by signs of oculosympathetic hyperactivity caused by irritation in the oculosympathetic pathway and shares etiologies with Horner Syndrome. We present the case of a 64-year-old woman with Pourfour du Petit syndrome due to compression of the second-order cervical sympathetic chain neuron from a dominant and prominent right internal jugular vein compensatory for contralateral agenesis. Internal jugular vein agenesis is a rare developmental vascular anomaly and is asymptomatic in the majority of patients with this condition.

## Introduction

Pourfour du Petit syndrome (PdPS) was first described by François Pourfour du Petit, a French anatomist, ophthalmologist, and neurologist, in 1727. He described mydriasis, eyelid retraction, and hemifacial hyperhidrosis in a soldier after a neck wound [1]. The syndrome comprises features opposite to Horner's syndrome, which is characterized by miosis, ptosis, and anhidrosis. Both syndromes are caused by disorders that disrupt the oculosympathetic system at any point between a three-neuron pathway. The first-order neuron originates from the posterolateral hypothalamus and descends through the brainstem and the lateral column of the cervical (C8) and thoracic (T1-T2) spinal cord. It synapses with the second-order preganglionic neuron that exits the spinal cord, passes under the subclavian artery, over the apex of the lung, and synapses with the third-order postganglionic neuron in the superior cervical ganglion at the level of the second cervical vertebra (C2). This third-order postganglionic neuron then branches. One branch travels along the external carotid artery to innervate the sudomotor and vasoconstrictor responses in the face. The other branch travels with the internal carotid artery to a further branch and innervates vasomotor responses and sweat glands of the forehead, the superior tarsal (Müller's) muscle of the eyelid, the orbital vasomotor and lacrimal gland, and the iris dilator muscle [2-4]. If a lesion irritates and hyperactivates any point along this oculosympathetic pathway, it leads to PdPS; however, if the lesion interrupts the pathway and causes oculosympathetic paresis, then Horner’s Syndrome develops. Thus, both syndromes share the same pathophysiology and causative etiologies with functionally opposite clinical manifestations.

We present a case report of second-order PdPS in a 64-year-old woman, secondary to irritation of the sympathetic cervical chain by a previously unreported vascular mechanism. Further, we will discuss unique practical clinical and diagnostic considerations in this rare syndrome.

## Case presentation

A 64-year-old woman with arterial hypertension on antihypertensive treatment with an angiotensin-converting enzyme inhibitor (enalapril) presented with a two-year history of transient episodes of right-sided hyperhidrosis, blurred vision, and photophobia triggered by Valsalva maneuvers. The episodes lasted approximately thirty to sixty seconds and subsided spontaneously with rest. Without an associated trauma history or identifiable trigger, the symptoms were noted to have become permanent over a two-week period, and the patient was referred for a neurological consultation.

The clinical examination revealed a right-sided mydriasis, exophthalmos, upper lid retraction, and unilateral right-sided internal jugular vein distention with no other abnormalities. A clinical diagnosis of second-order PdPS was made.

**Figure 1:**
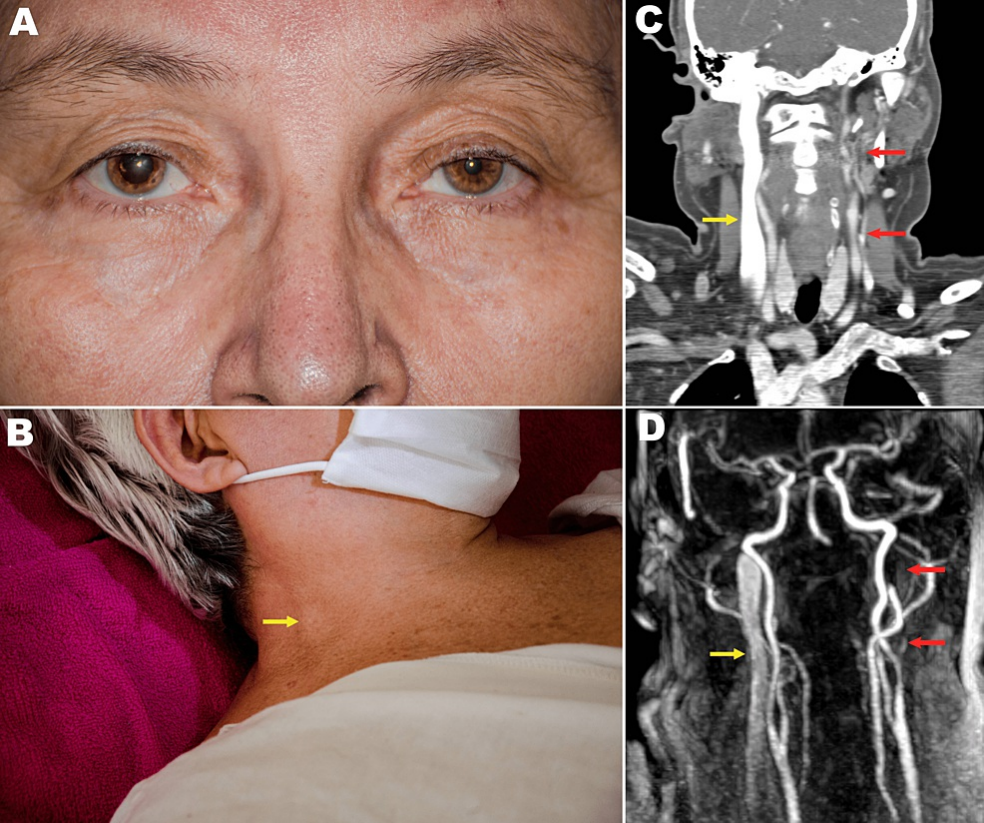
Images of the patient showing Pourfour du Petit syndrome. CT and MR angiography images are also seen. Right mydriasis, exophthalmos, and upper lid retraction (Panel A). Right-sided internal jugular vein distention (yellow arrows) on examination (Panel B), CT angiography (Panel C), and magnetic resonance angiography (Panel D). Red arrows highlight left internal jugular vein agenesis.

The patient's blood cell count, renal function, liver function, electrolytes, coagulation screen, thyroid hormone profile, and C-reactive protein were all within normal range. Neck and chest x-rays to assess for apical lung neoplasms and cervical ribs were unremarkable. A computed tomography (CT) of the neck and chest did not identify any causative cervical or thoracic infectious or neoplastic mass lesions. Magnetic resonance imaging (MRI) of the orbits and the brain was unremarkable. No evidence of breast cancer was found on breast examination or mammography. Finally, CT and MR angiographic imaging of the chest and neck revealed a left internal jugular vein agenesis and a right-sided internal jugular vein distention with potential vascular compression of the right cervical sympathetic chain (Figure 1). With angiographic imaging of the neck, the mechanistic etiology for PdPS in our patient was determined as congenital left internal jugular vein agenesis with compensatory enlargement of the right internal jugular vein causing compressive irritation and activation of second-order nerve fibers in the right oculosympathetic pathway.

The case was discussed with the patient, and surgical management was declined. She was discharged with a plan for periodic neurological, ophthalmological, and vascular evaluations.

## Discussion

PdPS resembles a reverse Horner syndrome and is clinically characterized by unilateral mydriasis, exophthalmos, and facial hyperhidrosis. The syndrome is the clinical expression of sympathetic cervical chain irritation, typically caused by mass effect or trauma, which leads to hyperstimulation of the ipsilateral oculosympathetic pathway. Known etiologies for PdPS include carotid dissection, cervical vertebral anomaly [5], lung, thyroid, and esophageal tumors; neck trauma [6], regional anesthesia [7], migraine and cluster headache [8,9], and iatrogenic injury [10].

PdPS can occur from an irritative lesion anywhere along the first, second, and third-order neurons of the oculosympathetic pathway. Given the broad spectrum of etiologies, the diagnostic approach must be directed by the suspected location of the lesion along the oculosympathetic pathway.

First-order PdPS is caused by a lesion in the central nervous system (CNS) that affects the posterolateral hypothalamus, dorsal mesencephalon, pons, medulla, or the spinal cord. Patients with first-order PdPS show ipsilateral hemihyperhidrosis with signs and symptoms of CNS damage [11] (contralateral hemiparesis, contralateral hypoesthesia, vertical gaze paresis, ataxic gait, cranial nerve palsies, vertigo, dysphagia, nystagmus, facial weakness, or in association with cluster headache [8]). In cases where a first-order PdPS is suspected, a head MRI or expedient non-contrast head CT should be performed to exclude CNS pathologies such as stroke, hemorrhage, or intracranial neoplasm. In cases where no lesion can be found, an intracranial CT or MR angiography must be taken to rule out an intracranial aneurysm.

As in our patient, a second-order PdPS manifests with unilateral ocular signs of sympathetic overactivity (mydriasis, exophthalmos, and upper lid retraction) and facial hyperhidrosis, due to the irritation of the preganglionic neuron between the spinal cord and the superior cervical ganglion. It can also present with arm and neck pain, brachial plexopathy, vocal cord paralysis, or even phrenic nerve palsy. The first step in our approach was to rule out a chest or neck neoplasia (particularly an apical lung mass, a paravertebral metastatic mass [2], breast cancer, neurofibroma, or thyroid adenoma), followed by a cervical rib and infections (tuberculosis, aspergillosis, and cryptococcosis [11] have been reported as causes of second-order PdPS). Next, to investigate potential known vascular etiologies, such as a thoracic aneurysm [12], we ordered chest and neck CT and MR angiography. In our angiographic studies, we found left-sided (contralateral) internal jugular vein agenesis and, as a result, right-side (ipsilateral) internal jugular vein distention that was compressing the ipsilateral sympathetic chain. These findings fit with the history of transient PdPS episodes triggered by Valsalva maneuvers of right-sided hyperhidrosis, blurred vision, and photophobia that then became permanent two weeks prior to presentation and neurological assessment. No direct or indirect signs of internal jugular vein thrombosis were found in the MR or CT angiography.

In third-order PdPS, the lesion is located in the postganglionic neuron, and the patient presents with mydriasis, exophthalmos, and upper lid retraction but not hyperhidrosis. Etiologies of a third-order syndrome include internal carotid artery lesions [2] (dissection, aneurysms, acute thrombosis, fibromuscular dysplasia, and arteritis), as well as non-vascular lesions of the neck, including enlarged lymph nodes, neck trauma [3], and cluster headaches [8]). Therefore, if a third-order syndrome is diagnosed, head and neck MR angiography must be done.

Treatment of the underlying condition usually resolves the symptoms. Sympathectomy is available for hyperhidrosis and the reduction of conjunctivitis and keratitis associated with exophthalmos. If surgical management is not indicated or refused, then ongoing neurological, ophthalmological, and vascular follow-up assessments are indicated to reduce the risk of ocular complications.

Developmental vascular anomalies occur in about 0.05% to 0.25% of the general population. Within these anomalies, agenesis of the internal jugular vein is extremely rare. Jugular vein agenesis is typically asymptomatic, though it can present as a contralateral, painless cervical mass [13]. It has been reported as an incidental finding in adult patients during attempted ultrasound-guided internal jugular vein central line placement and during preoperative assessments for major surgical procedures [14]. When investigating potential vascular malformations, a prior study of patients with neck and facial venous malformations found that 20% also had cerebral developmental venous anomalies [15]. Thus, the presence of peripheral and superficial vascular malformations of the head and neck merits consideration of intracranial angiographic studies to rule out vascular abnormalities in the brain.

Our case represents a previously unreported mechanism for second-order PdPS in which the sympathetic cervical chain on the clinically affected side is compressed by a distended ipsilateral internal jugular vein resulting from agenesis of the contralateral internal jugular vein. This further highlights the diagnostic role of angiographic studies in PdPS patients of unclear etiology.

## Conclusions

Internal jugular vein agenesis is a rare congenital vascular anomaly that is typically asymptomatic but can present with contralateral PdPS due to compensatory contralateral internal jugular vein distention. It is important to investigate arterial and venous abnormalities, including developmental vascular anomalies, in every patient with second-order PdPS in whom neoplastic, traumatic, and infectious etiologies have been ruled out.
